# Modified Chitosan for Silver Recovery—Kinetics, Thermodynamic, and Equilibrium Studies

**DOI:** 10.3390/ma13030657

**Published:** 2020-02-01

**Authors:** Bogdan Pascu, Cristina Ardean, Corneliu Mircea Davidescu, Adina Negrea, Mihaela Ciopec, Narcis Duțeanu, Petru Negrea, Gerlinde Rusu

**Affiliations:** 1 Faculty of Industrial Chemistry and Environmental Engineering, Politehnica University of Timisoara, 2 Piata Victoriei, RO 300006 Timisoara, Romania; ioan.pascu@upt.ro (B.P.); cristina.ardean@sudent.upt.ro (C.A.); adina.negrea@upt.ro (A.N.); mihaela.ciopec@upt.ro (M.C.); narcis.duteanu@upt.ro (N.D.); gerlinde.rusu@upt.ro (G.R.); 2 Research institute for renewable energy, 138, Gavril Musicescu Street, 300777 Timisoara, Romania

**Keywords:** silver recovery, adsorption, chitosan, functionalization

## Abstract

The aim of this study is to investigate the silver recovery from aqueous solutions. There are a variety of recovery methods, such as hydrometallurgical, bio-metallurgical, cementation, reduction, electrocoagulation, electrodialysis, ion exchange, etc. Adsorption represents a convenient, environment friendly procedure, that can be used to recover silver from aqueous solutions. In this paper we highlight the silver adsorption mechanism on chitosan chemically modified with active groups, through kinetic, thermodynamic, and equilibrium studies. A maximum adsorption capacity of 103.6 mg Ag(I)/g of adsorbent for an initial concentration of 700 mg/L was noticed by using modified chitosan. Lower adsorption capacity has been noticed in unmodified chitosan—a maximum of 75.43 mg Ag(I)/g. Optimum contact time was 120 min and the process had a maximum efficiency when conducted at pH higher than 6. At the same time, a way is presented to obtain metallic silver from the adsorbent materials used for the recovery of the silver from aqueous solutions.

## 1. Introduction

In recent years, many applications have been developed in which silver nanoparticles are used, as a result of its antimicrobial properties. A consequence of these applications are the significant quantities of aqueous solutions with residual silver ions that are formed [1].

Around 79 part per billion [2] of earthly bark is made of silver, a precious metal. The value of silver has been known for over 6000 years, having a significant role in different moments of human life [3,4,5]. The demand for silver has progressively increased, with the development of the electrical and electronic industry, but also because it is cheaper than gold or platinum [6]. In the last years, the natural resources for silver have decreased, which is why the cost of silver production has increased rapidly, despite its market price, which is declining; some of the known applications of silver are: photographic industry, radiology, electronics, batteries, jewelry, dental materials, biomedical materials, medicines, water disinfection or wastewater treatment [4,7,8,9]. A variety of methods for silver recovery from aqueous solutions have been developed, but adsorption is the most efficient method with low energy consumption [10]. Adsorption is a process that takes place on the surface of the material, when ions, molecules, or atoms adhere to the surface of the substrate through physical, physical–chemical, or chemical interactions [11]. Also, a large number of materials with adsorbent properties have been developed, but a special attention is paid to biomaterials such as agricultural waste [12]. Chitosan is a biomaterial with good adsorbent properties [13]. Chitosan (poly-β-(1→4)-2-amino-2-deoxy-D-glucose) is a nitrogenous (amino-based) polysaccharide, with a macromolecular structure, non-toxic, biocompatible, biodegradable, and inexpensive [14]. Chitosan is a material that presents some adsorbent properties [15,16,17,18,19,20,21,22,23,24,25,26,27] and that can be easily characterized using physical–chemical methods [28]. In order to improve the adsorbent properties of chitosan, it can be functionalized with pendant groups. In the present paper, the recovery mechanism of metallic silver from aqueous solutions was studied and the adsorbent properties of chitosan were compared to functionalized chitosan with phosphonium groups. The adsorption mechanism has been highlighted by kinetic, thermodynamic, and equilibrium studies.

## 2. Experimental Part

### 2.1. Materials Synthesies

**Figure 1:**
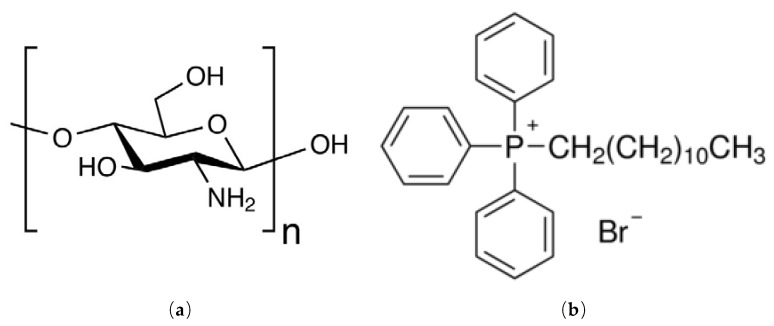
The structure of: (**a**) chitosan (Ch), and (**b**) dodecyl-triphenyl-phosphonium bromide (DDTPPBr).

In order to obtain the adsorbent material, the following were used: Chitosan (Ch) (Figure 1a) as solid support, and dodecyl-triphenyl-phosphonium bromide (DDTPPBr) as extractant, known by the pendant groups (P) presented in structure, which can be seen in Figure 1b.

The method used for the chemical modification of chitosan, by impregnation, was the SIR (solvent impregnated resin) method [29]; the extractant, dodecyl-triphenyl-phosphonium bromide (DDTPPBr) was dissolved in water, and then added to the solid support (chitosan), with a mass ratio solid support:extractant = 10:1. 

For functionalization by impregnation, the solid support and the extractant were left in contact for 24 h. After the contact time elapsed, the samples were filtered, washed with distilled water, and dried at 50 °C for 24 h.

### 2.2. Material Characterization

Obtained material was characterized by scanning electron microscopy (SEM) and X-ray energy dispersion (EDX, FEI, Hillsboro, Oregon, SUA) using a FEI Quanta FEG 250i scanning electron microscope (FEI, Hillsboro, Oregon, SUA), and Fourier-transform infrared spectroscopy (FTIR, Bruker, Billerica, Massachusetts, SUA), using a spectrometer Platinum ATR-QL Diamond, in the range 4000–400 cm^−1^.

### 2.3. Batch Adsorption Experiments 

To highlight the need for functionalization via impregnation of chitosan, the silver recovery studies were performed comparatively on both the obtained Ch-DDTPPBr and on the unfunctionalized chitosan. 

The adsorption studies were carried out in a water bath Julabo SW23 (Julabo, Seelbach, Baden-Württemberg, Germany), with a thermostat and shaking speed of 200 rpm.

In order to determine the mechanism of the adsorption process, the adsorption capacity of the material q (mg/g) was determined using the following equation:

(1) $$q=\frac{\left(C_{0}-C_{f}\right)V}{m},$$

where:

C_o_—the initial concentration of silver (I) in solution, (mg/L)
C_f_—the residual concentration of silver (I) in solution, (mg/L)
V—the volume of solution, (L)
m—mass of the adsorbent material, (g)

The influence of pH, contact time, temperature, and the initial concentration have been studied, by determining the influence over the adsorption capacity of the material.

Thus, in order to evaluate the influence of pH onto the adsorption capacity, samples of ~0.1 g of adsorbent material were weighed, over which 25 mL of AgNO_3_ solution containing 10 mg Ag/L with pH values in the range of 1–12 was added. The pH of the solution was adjusted with buffer solutions and measured using an METTLER TOLEDO Seven Compact S210 pH meter (Mettler Toledo, Columbus, Ohio, SUA). The samples were kept in contact for 120 min, then filtered and the residual concentration of Ag (I) in the solutions was determined by atomic absorption spectrometry, using a Varian SpectrAAS 280 FS atomic absorption spectrophotometer (Varian, Palo Alto, California, SUA). To determine the influence of the contact time and the temperature onto the adsorption process, the contact time was varied between 30 and 240 min, at three temperatures (298 K, 308 K, and 318 K). Studies were carried out with an initial solution of Ag (I) of 10 mg/L at pH ~2. The amount of material used was ~0.1 g and the residual concentration of Ag (I) in the solutions was determined by atomic absorption spectrometry. To determine the equilibrium concentration and the effect of the initial concentration of Ag (I) on the adsorption capacity of the material, solutions of Ag (I) with different concentrations were prepared (10, 50, 75, 100, 150, 200, 300, 400, 500, and 600 mg/L) for chitosan and for the Ch-DDTPPBr (10, 50, 75, 100, 150, 200, 300, 400, 500, 600, 700, 800, and 900 mg/L). These were obtained by dilution from a stock solution of 1000 mg/L AgNO_3_. Adsorption was performed at a pH ~2 for 1 h and at 298 K.

Two kinetic models were used for the kinetic studies: the pseudo-first-order, proposed by Lagergren [30] and the pseudo-second-order kinetic model, proposed by Ho and McKay [31].

The thermodynamic parameters: Gibbs free energy (ΔG°), enthalpy (ΔH°), and the entropy (ΔS°). At the same time, the activation energy was determined, E_a_ were established [32]. 

To determine the maximum adsorption capacity of the Ch-DDTPPBr material comparatively with Ch, the experimental data was modeled using the Langmuir [33], Freundlich [34], and Sips isotherm [35]. 

### 2.4. Silver Recovery 

After the adsorbent is exhausted, it will contain a considerable amount of Ag (I). For the recovery of Ag (I), the exhausted material was decomposed at 600 °C, for 240 minutes, with a heating speed of 5 °C /min, using a controlled atmosphere furnace (Nabertherm LHT407GN Furnaces, Nabertherm, Lilienthal, Germany). The sample obtained after the decomposition was analyzed through scanning electron microscopy (SEM, FEI, Hillsboro, Oregon, SUA) and X-ray energy dispersion (EDX) using a FEI Quanta FEG 250 scanning electron microscope (FEI, Hillsboro, Oregon, SUA).

## 3. Results and Discussions 

### 3.1. Characterization of the Synthesized Materials 

#### 3.1.1. Scanning Electron Microscopy (SEM) and X-Ray Energy Dispersion Spectroscopy (EDX).

**Figure 2:**
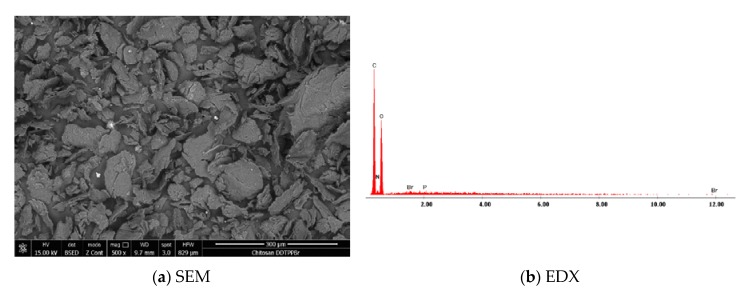
Scanning electron microscopy of new produced adsorbent material: (**a**) SEM and (**b**) X-ray energy dispersion—EDX.

To highlight that the adsorbent (Ch) was functionalized through impregnation with the DDTPPBr extractant, obtained material was characterized by scanning electron microscopy, SEM (Figure 2a), and X-ray energy dispersion spectroscopy, EDX (Figure 2b). From the EDX analysis we can observe characteristic peaks of the specific elements of the support, C, N, and O and also peaks characteristic to the extractant (DDTPPBr (P)).

#### 3.1.2. Fourier-Transform Infrared Spectroscopy, FT-IR

The main objective of the FT-IR spectroscopy is to determine the functional groups of the two materials, but in particular it highlights the specific extractant DDTPPBr specific groups, used to functionalize by impregnation the support, Ch.

From the FT-IR spectra of the two materials, we can observe similarities, such as: around the wavelength of 3440 cm^−1^ was observed a band that is specific to the stretching vibration of the O–H group, followed by the vibrations specific to the aliphatic group –C–H which occurs at 2900 cm^−1^. Around the wavelength of 1600 cm^−1^ the peak specific to the vibrations of the C=O group was observed and at the wavelength of 1500 cm^−1^ appears a specific peak of the N–H bond. All of these vibrations are specific for the solid support, Ch [36]. 

**Figure 3:**
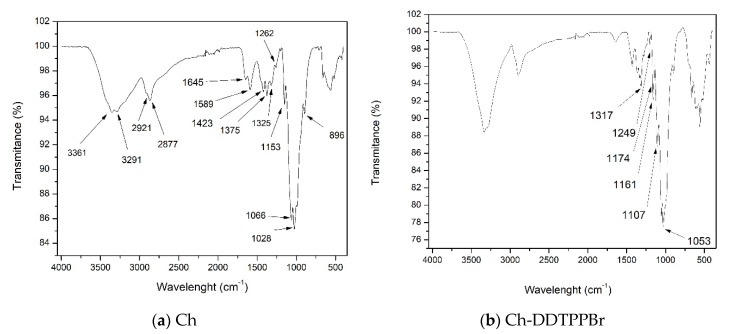
Fourier-transform infrared spectroscopy, FT-IR for the materials: (**a**) Ch and (**b**) Ch-DDTPPBr.

From the Ch-DDTPPBr FT-IR spectrum presented in Figure 3b, two peaks can be seen around the wavelength of 1249 cm^−1^ and 1317 cm^−1^ which are specific to the P=O group noticed in the extractant (dodecyl-triphenyl-phosphonium bromide) that functionalized the chitosan. Also, the peaks corresponding to the wavelengths of 1053 cm^−1^, 1107 cm^−1^, 1161 cm^−1^, and 1174 cm^−1^ are specific to the vibration of the P–O–C bond present in aryl phosphate compounds [37].

### 3.2. Adsorption Studies

#### 3.2.1. Influence of the pH on the Adsorption Process

**Figure 4:**
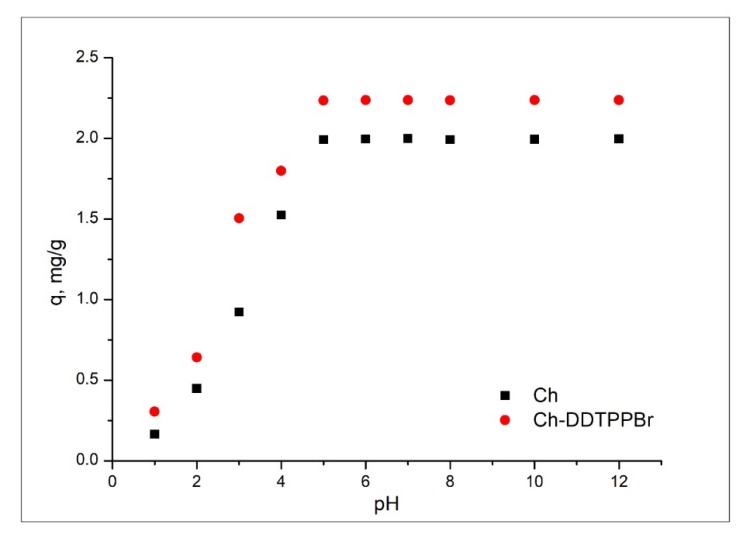
The influence of pH on the adsorption process.

It is known that the pH is one of the most important factor that influences the adsorptive processes. This is due to the influence of the H^+^ concentration in the solution, which can influence the chemistry of the Ag (I) solution, as well as the protonation of the present functional groups, such as phenyl, or phosphonium. Figure 4 shows the influence of the pH of the aqueous solution with Ag (I) ions on the adsorption capacity of the materials. 

It can be seen from Figure 4 that the adsorption of Ag (I) is influenced throughout the studied pH range, 2–12, but it can be observed that at pH > 5 the active groups have been protonated and are available to be easily complexed by Ag (I) ions [38,39].

#### 3.2.2. The Influence of the Time of Contact and Temperature on the Adsorption Process

**Figure 5:**
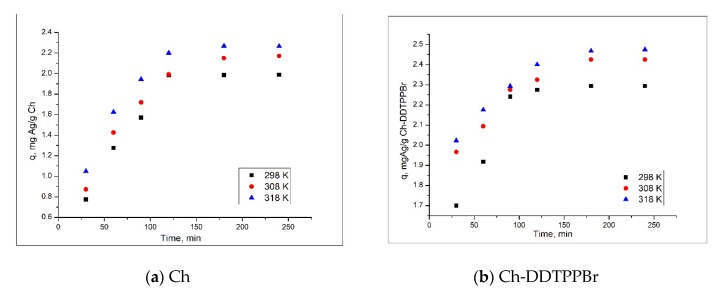
Contact time and temperature influence (**a**) Ch; (**b**) Ch-DDTPPBr.

In adsorption processes, contact time, and temperature are the two important parameters. Thus, the influence of the contact time and the temperature on the adsorption capacity of the studied materials is presented in Figure 5.

From the experimental data it is observed that along with increase of the contact time, the adsorption capacity of the two materials also increases, until it reaches a certain time of contact (~120 min), after which the adsorption capacity remains approximately constant. Also, it is noted that the material Ch-DDTPPBr has a higher adsorption capacity than that of Ch, ~2.4 mg/g compared to ~2 mg/g. It is also noticed that with the increase of temperature, the adsorption capacity of the materials also increases, but it is insignificant, economically speaking. 

### 3.3. Kinetic Studies

For the kinetic studies, two kinetic models were used: pseudo first-order proposed by Lagergren [30] and the pseudo second-order kinetic model, proposed by Ho and McKay [31]. The pseudo first-order equation can be expressed as follows:

(2) $$\frac{{\mathrm{dq}}_{t}}{\mathrm{dt}}=k_{1}\left(q_{e}-q_{t}\right),$$

where: q_e_ and q_t_ are the adsorbed amounts of silver per unit mass of Ch-DDTPPBr at equilibrium and time t respectively, and k_1_ is the adsorption rate constant for pseudo first-order adsorption. 

The q_t_ at different times t can be determined by the following pseudo first-order kinetic equation after integrating:

ln (q_e_ − q_t_) = ln q_e_ − k_1_t. (3)

The pseudo second-order kinetic model can be presented with the following equation:

(4) $$\frac{{\mathrm{dq}}_{t}}{\mathrm{dt}}=k_{2}{\left(q_{e}-q_{t}\right)}^{2},$$

where k_2_ is the rate constant for the pseudo second-order adsorption.

By linearizing this equation, we obtain:

(5) $$\frac{t}{q_{t}}=\frac{1}{k_{2}q_{e}^{2}}+\frac{t}{q_{e}}.$$

The linear variants of the two models are used for the modeling of the experimental data.

**Figure 6:**
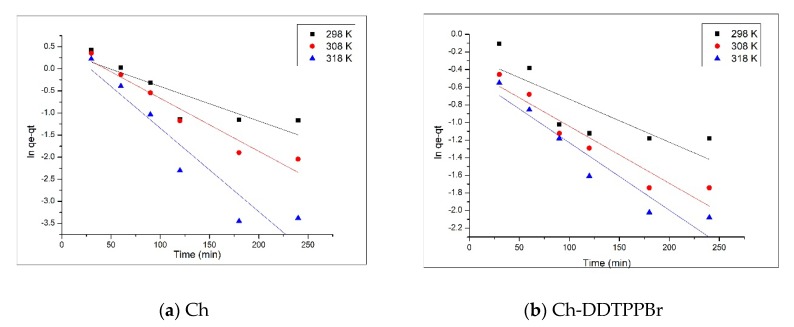
Pseudo first-order kinetic model (**a**) Ch; (**b**) Ch-DDTPPBr.

**Figure 7:**
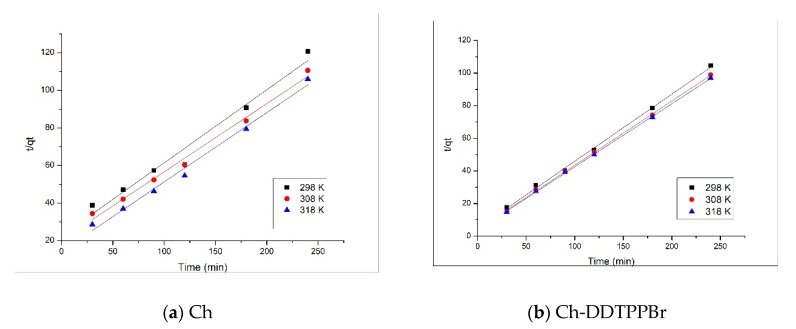
Pseudo second-order kinetic model (**a**) Ch; (**b**) Ch-DDTPPBr.

The velocity constant for the pseudo first-order kinetic model is determined from the linear representation of ln(q_e_ − q_t_) versus time (Figure 6a and Figure 7a), and the velocity constant for the pseudo second-order is estimated from the linear representation of t/q_t_ versus time (Figure 7a,b). Depending on the values of the constants and the regression coefficients (R^2^) obtained (Table 1), the kinetic model that best describes the adsorption process can be established.

Analyzing the kinetic parameters associated with the pseudo first-order kinetic model, presented in Table 1, and more precisely the regression coefficient, which presents values far from 1, being between 0.76 and 0.90 for Ch, and between 0.86 and 0.91 for Ch-DDTPPBr, it can be stated that this model does not accurately describe the adsorption process of Ag (I) on the two studied materials. Simultaneously, based on the pseudo first-order kinetic model the adsorption capacities were also evaluated (q_e_, q_calc_), whose values differ greatly from the experimentally obtained results (q_e_, q_exp_). 

Subsequently, the experimentally obtained data were modeled using the pseudo second-order kinetic model, in order to establish if this model better describes the adsorption process. The linearized forms of the pseudo second-order kinetic model graphically represented in Figure 7a,b are obtained by graphically representing the dependence t/q_t_ versus time for the two studied materials at three different temperatures. Analyzing the kinetic parameters presented in the previous table and more precisely the regression coefficient R^2^, which is very close to a unit value (1), it can be stated that the pseudo second-order kinetic model describes very well the adsorption process of Ag (I). Also, in support of this affirmation comes the fact that, the values obtained from the calculation of the adsorption capacity (q_e_, q_calc_), are very close to the experimentally obtained values (q_e_, q_exp_). This is based on the hypothesis that in the process of Ag (I) recovery on both of the materials, the determining step is a physical process and takes place through the formation of physical links between the substrate and Ag (I) ions [31,40]. 

### 3.4. Thermodynamic Studies

The effect of the temperature on the adsorption process of Ag (I) on Ch and Ch – DDTPPBr was also studied. As the temperature increases, the adsorption capacity of the material increases, so we can say that the process is endotherm. Specific thermodynamic parameters: Gibbs free energy (ΔG^0^), free enthalpy (ΔH^0^), and the free entropy (ΔS^0^) were calculated with the following relations:

ΔG^0^ = −RTlnK_d_, (6)

where,

(7) $$K_{d}\;=\;\frac{C_{\mathrm{Ae}}}{C_{e}},$$

(8) $$\log\;K_{d}\;=\;\frac{∆S^{0}}{2.3\;R}-\frac{∆H^{0}}{2.303\;\mathrm{RT}}.$$

where:

*R* is the gas constant,
*K*_d_ is the equilibrium constant,
*T* is the temperature (K),
*C*_Ae_ is the equilibrium concentration Ag(I) on adsorbent (mg/L), and
*C*_e_ is the equilibrium concentration of Ag(I)in the solution (mg/L).

**Figure 8:**
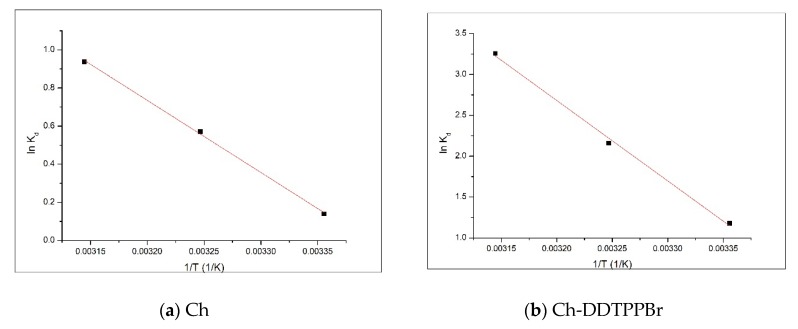
Van’t Hoff plots for the adsorption of Ag (I) onto (**a**) Ch and (**b**) Ch-DDTPPBr.

The thermodynamic parameters for the studied adsorption process are evaluated from the slope and intercept of linear dependence of lnK_d_ vs. 1/T, plot shown in Figure 8. Values of thermodynamic parameters obtained for Ag(I) adsorption on the studied materials are presented in Table 2.

The negative values of the Gibbs free energy suggest that the adsorption of Ag(I) on the studied materials occurs spontaneously. Also, the decrease of the Gibbs free energy with temperature decrease shows that the adsorption process of Ag (I) is favored by higher temperatures. The positive values of the standard enthalpy variation confirm that the process is endothermic; this fact is also supported by the slight increase of the adsorption capacity at equilibrium and the pseudo second-order velocity constant (k_2_) with the increase of the temperature. According to the data from the literature, if ΔH° is > 20 kJ/mol, the process can be considered of a physical–chemical nature, for Ch and of chemical nature for Ch-DDTPPBr, having ΔH° > 40 KJ/mol [39]. The standard entropy variation has positive values which suggests that the adsorption causes a higher disorder at the liquid/solid interface. However, the values of the standard entropy variation are small, indicating that no major changes occur.

### 3.5. Activation Energy

Activation energy E_a_ was calculated using the Arrhenius equation and the velocity constant of the pseudo second-order kinetic model (k_2_), constant which is specific to the adsorption process of the metal ions on the obtained materials by chemically modifying the support through functionalization with pendant groups.

lnk_2_ = lnA − Ea RT, (9)

where:

k_2_—velocity constant (g/min∙mg)
A—Arrhenius’s constant (g∙min/mg)
Ea—activation energy (kJ/mol)
T—absolute temperature (K)
R—ideal gas constant (8.314 J/mol∙K)

**Figure 9:**
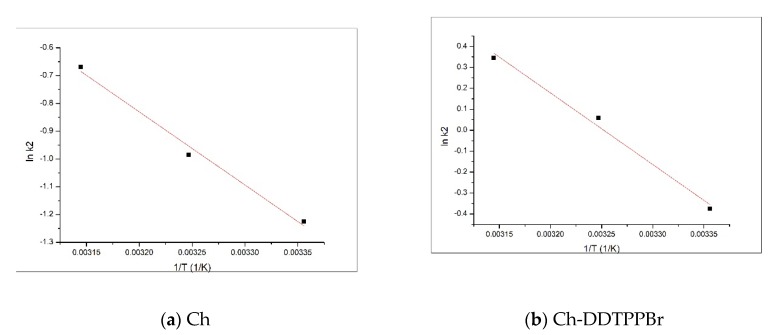
Activation energy (**a**) Ch; (**b**) Ch-DDTPPBr.

The activation energy of the adsorption of different metals on the functionalized support is calculated from the equation of the graphical representation of ln k_2_ versus 1/T (Figure 9).

For the studied adsorption processes, the activation energy has a value of 21.8 kJ/mol for the adsorption of Ag (I) on Ch, with a correlation coefficient of 0.9904, and 28.4 kJ/mol for the adsorption of Ag (I) on the Ch-DDTPPBr with a correlation coefficient of 0.9925. Based on the value of the activation energy it can be concluded that the adsorption Ag (I) on the studied materials is a physical adsorption because the value of the activation energy is bigger than 8 kJ/mol [41,42].

### 3.6. Equilibrium Studies—Adsorption Isotherms

The adsorption isotherms are very important for the analysis of the adsorption process. In order to describe the adsorption mechanism of Ag (I) on the Ch and Ch-DDTPPBr materials, Freundlich, Langmuir, and Sips models were used.

Langmuir isotherm is applied for adsorption on homogeneous surfaces [43,44,45]. The nonlinear expression of Langmuir’s equation isotherm [33] can be expressed as follows:

(10) $$q_{e}=\;\frac{q_{max}K_{L}C_{f}}{1+\;K_{L}C_{f}},$$

where:

*q_e_*—the maximum absorption capacity (mg/g)
*C_f_*—the equilibrium concentration or final concentration of Ag(I) in solution (mg/L)
*q_max_*—Langmuir maximum adsorption capacity (mg/g)
*K_L_*—Langmuir constant.

The Freundlich isotherm can be applied to heterogeneous adsorption surface [44,46]. The nonlinear form of the Freundlich isotherm equation [34] is:

(11) qe= KFCf1nf,

where:

*q_e_*—the maximum absorption capacity (mg/g)
*C_f_*—the equilibrium concentration or final concentration of Ag (I) in solution (mg/g)
*K_F_* and *n_f_*—the characteristic constants that can be related to the relative adsorption capacity of the adsorbent and the intensity of adsorption.

The Sips isotherm is a combined form of the two models previously presented. The nonlinear expression of the Sips isotherm [35] is:

(12) qe= qsKsCe1ns1+ KSCe1ns,

where:

*q_S_*—the maximum absorption capacity (mg/g)
*K_S_*—constant related to the adsorption capacity of the adsorbent
*n_S_*—the heterogeneity factor.

**Figure 10:**
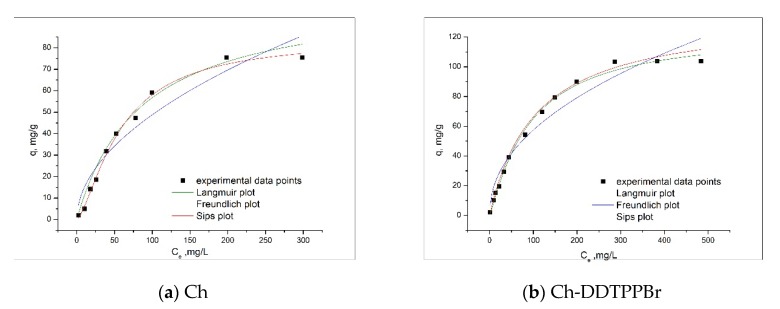
Isotherm model for adsorption of Ag (I) on the adsorbent materials (**a**) Ch; (**b**) Ch-DDTPPBr.

Figure 10 presents the three equilibrium isotherms, and Table 3 presents the parameters of the equilibrium isotherms for the adsorption of Ag (I) on the two adsorbent materials.

It can be noted that, along with the increase of the initial concentration of the Ag (I) solution, the maximum adsorption capacity of studied adsorbent material increases. So, for Ch material it is q_m_,_exp_ = 75.34 mg/g for an initial Ag (I) concentration of 400 mg/L, and for Ch-DDTPPBr q_m,exp_ = 103.6 mg/g for an initial Ag (I) concentration of 700 mg/L. From the obtained data it is observed that the highest adsorption capacities were obtained with the Ch-DDTPPBr material, which confirms that the functionalized chitosan with pendant groups considerably raises on the qualities of the adsorbent material.

The values of the heterogeneity factor 1/nf are between 0.4 and 0.51 and their deviation from the value of 1 indicates that the surface of the obtained adsorbent material is heterogeneous. The data from Table 3 indicates that for the adsorption of Ag (I), regardless of the nature of the used adsorbent material, the Freundlich isotherm has the lowest regression coefficient (R^2^), suggesting that this model is not accurately describing the adsorption process of Ag onto studied chitosan materials. The Sips model has higher values for the regression coefficient (R^2^). This fact indicates that this isotherm best correlates with the experimental data.

Table 4 presents a comparison between the maximum adsorption capacities obtained for silver recovery when different materials were used as adsorbents. Based on the data presented in Table 4 it can be noticed that the new produced material (Ch-DDTPPBr) represents a useful adsorbent for silver recovery from diluted solutions.

### 3.7. Silver Recovery 

**Figure 11:**
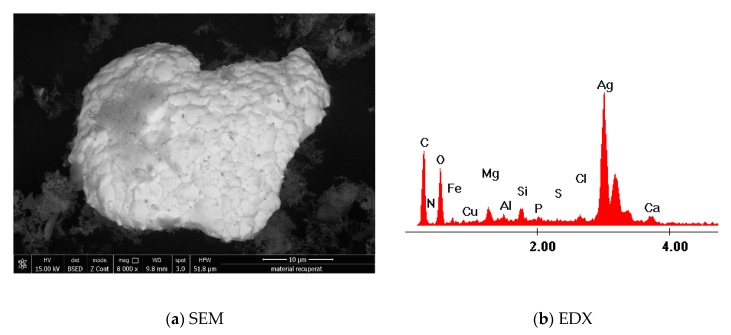
Scanning electron microscopy and X-ray energy dispersion in order to highlight the obtaining of metallic silver (**a**) SEM; (**b**) EDX.

After adsorption process, the used adsorbent material with silver content was the subject of a thermal decomposition carried out in controlled atmosphere at 600 °C for 240 min. Such thermal treatment has been carried out in order to recover metallic silver from exhausted material. The sample obtained after the decomposition of the exhausted Ch-DDTPPBr was characterized by scanning electron microscopy (SEM) (Figure 11a) and X-ray energy dispersion (EDX) (Figure 11b).

The images obtained by SEM provide information regarding the particle morphology and the distribution of the silver particles in the ash. From analysis of recorded SEM pictures it has been observed that the silver particles obtained after the thermal decomposition present a relative uniform distribution in the ash. The EDX analysis highlights the presence of silver in the resulted ash, after the decomposition of the exhausted material. The other elements in the composition of the ash along with silver are ash-specific elements. Metallic silver was further recovered from ash by leaching in order to remove all other components.

From the presented data it can be concluded that the silver can be recovered from the exhausted material.

## 4. Conclusions

The experimental results, at a laboratory scale, obtained in this study showed that the new obtained material through functionalization of chitosan with the active groups of dodecyl-triphenyl-phosphonium bromide, DDTPPBr, shows an increased efficiency for the removal of Ag (I) from aqueous solutions, compared to the unfunctionalized chitosan. The maximum adsorption capacity of the material was 103.6 mg Ag (I)/g, for a maximum concentration of Ag (I) of 700 mg/L, compared to unfunctionalized chitosan, which has a maximum adsorption capacity of 75.34 mg Ag (I)/g. The contact time required for the adsorption process was 120 min. The process runs with maximum efficiency at a pH > 6. The adsorption mechanism is also supported by the kinetic, thermodynamic, and equilibrium studies. Thus, the adsorption process is subjected to pseudo second-order kinetics, and the isotherm that best describes the adsorption is the Sips isotherm. Adsorptive process is spontaneous, and the adsorption is carried out by physical interactions between the metal ion and the active centers of the material, in the case of Ch-DDTPPBr and physical–chemical interactions for the unfunctionalized Ch. 

The proposal for the recovering process of the metallic silver from the exhausted material is another target of this study. Metallic silver can be recovered through incinerating the exhausted material at 600 °C, which then can be used in different industrial fields such as: electronics, medicine, dentistry, jewelry, chemical industry or to obtain materials with anti-corrosive properties, etc.

## Figures and Tables

**Table 1:** Kinetic parameters for adsorption of Ag (I) on the adsorbent materials.

Parameter	q_e_,_exp_, mg/g	Pseudo-first Order			Pseudo-second Order		
Temp., K		q_e_,_calc_, mg/g	k_1_, min^−1^	R^2^	q_e_,_calc_, mg/g	k_2_, min^−1^(mg/g)^−1^	R^2^
**Chitosan (Ch)**							
298	1.98	1.46	0.0078	0.7691	2.04	0.2937	0.9739
308	2.17	1.70	0.0120	0.9415	2.30	0.3734	0.9909
318	2.26	1.72	0.0019	0.9030	2.32	0.5124	0.9905
**Ch-DDTPPBr**							
298	2.29	1.28	0.0049	0.8663	2.43	0.6877	0.9981
308	2.43	1.44	0.0067	0.9019	2.53	1.0604	0.9996
318	2.46	1.25	0.0079	0.9187	2.63	1.4135	0.9997

**Table 2:** Thermodynamic parameters for adsorption of Ag(I) on the adsorbent materials.

Materials	ΔH°, kJ/(mol)	ΔS°, kJ/(mol·K)	ΔG°, kJ/mol			R^2^
			298 K	303 K	308 K	
Ch	31.4	0.106	−0.36	−1.42	−2.24	0.9993
Ch-DDTPPBr	81.8	0.284	−2.84	−5.68	−8.52	0.9974

**Table 3:** Parameters of isotherm model for adsorption of Ag(I) on the adsorbent materials.

Materials	q_m,exp_, mg/g	Freundlich Isotherm			Langmuir Isotherm			Sips Isotherm			
		K_F_, mg/g	1/n_f_	χ^2^	K_L_, L/mg	q_L_, mg/g	χ^2^	K_s_	q_s_, mg/g	1/n_s_	χ^2^
Ch	75.34	4.59	0.51	1.51	0.011	100.8	0.02	0.0026	85.40	3.9	0.13
Ch-DDTPPBr	103.6	6.81	0.46	1.69	0.009	135.8	0.01	0.0061	113.7	2.1	0.08

**Table 4:** Comparison of maximum adsorption capacities obtained for different adsorbents.

Absorbents	Maximum Adsorption Capacities	References
CMC/CMC_TS_ and SSS powder hydrogel	0.451 mg Ag(I)/g	[38]
Grapefruit peels, GP	10.92 mg Ag(I)/g	[47]
Grapefruit peels modified with urea, GPU	66.83 mg Ag(I)/g	[47]
Grapefruit peels modified with melamine, GPM	28.05 mg Ag(I)/g	[47]
Biosolids biochar	43.9 mg Ag(I)/g	[1]
Stillage residue biochar	23.0 mg Ag(I)/g	[48]
Coconut shell activated carbon	55.0 mg Ag(I)/g	[49]
Chitosan, Ch	75.34 mg Ag(I)/g	This paper
Chitosan functionalized with dodecyl-triphenyl-phosphonium bromide, Ch-DDTPPBr	103.6 mg Ag(I)/g	This paper
